# Supplementary figures and images for: p97/UBXD1 Generate Ubiquitylated Proteins That Are Sequestered into Nuclear Envelope Herniations in Torsin-Deficient Cells
Source: Int J Mol Sci. 2022 Apr 21;23(9):4627. doi: 10.3390/ijms23094627 (PMC9100061; doi:10.3390/ijms23094627)

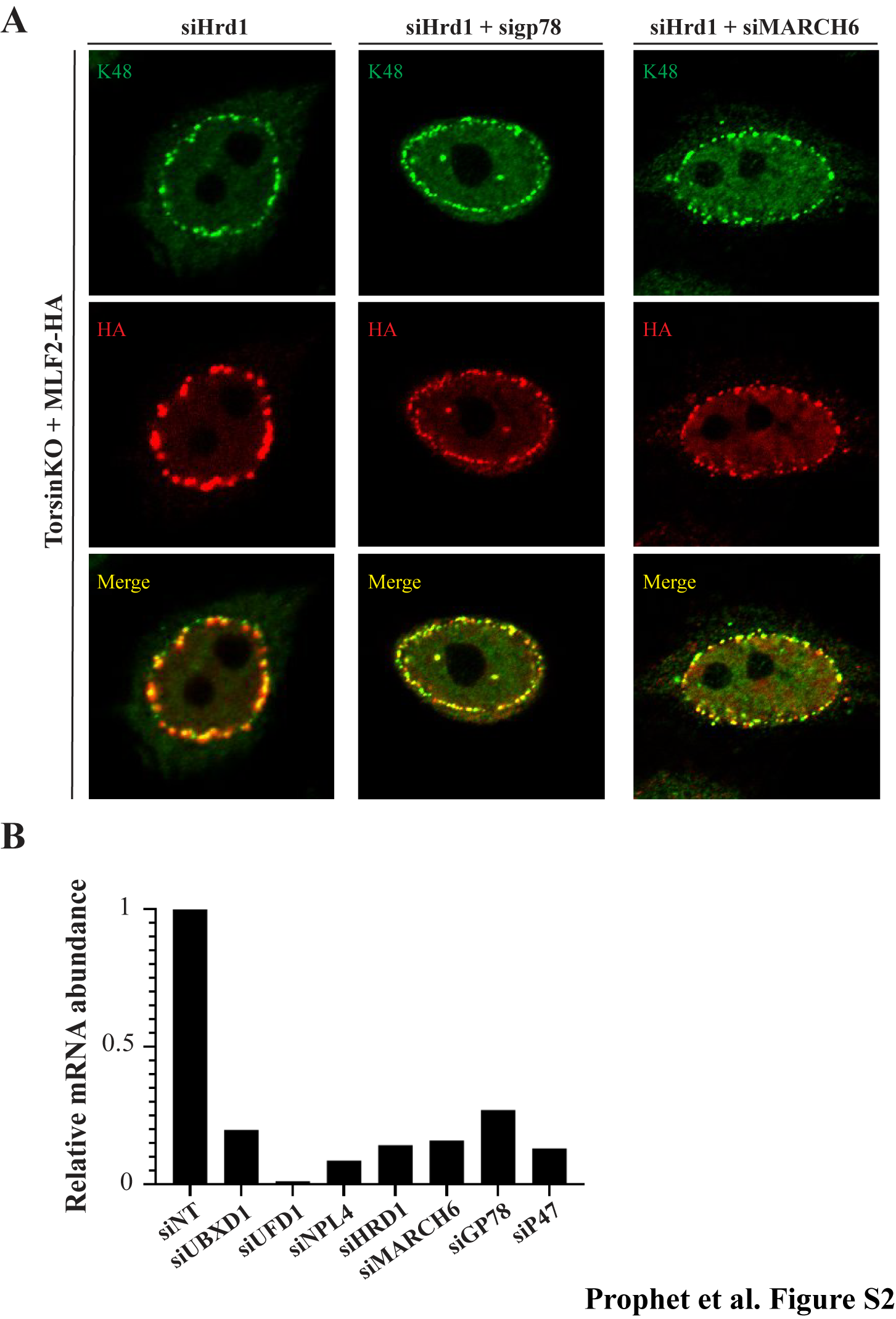

Supplement: Supplementary file 1 [file ijms-23-04627-s001.zip › Fig. S2- Translocon KD v2.tif]

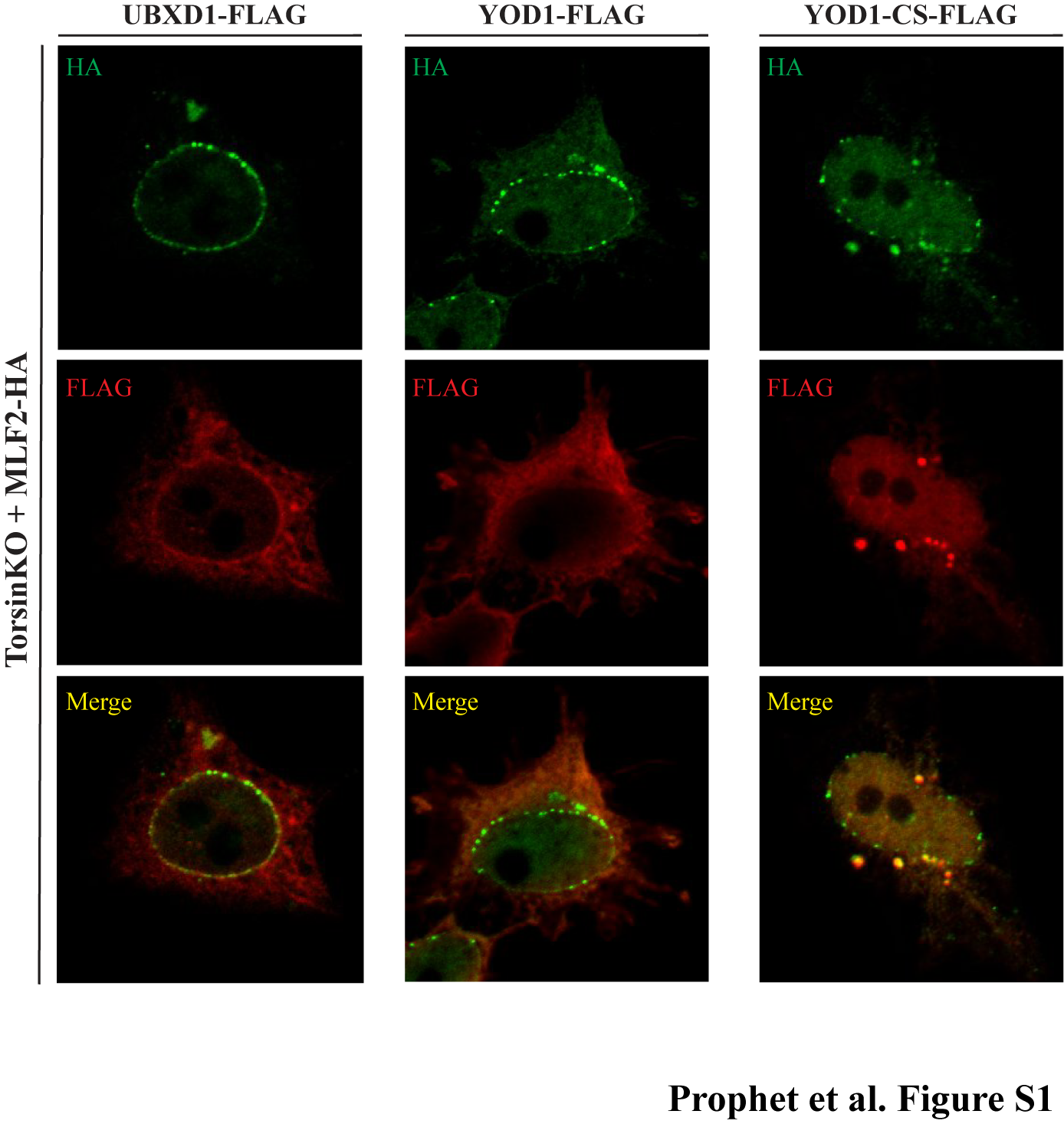

Supplement: Supplementary file 1 [file ijms-23-04627-s001.zip › Fig. S1- Localization.tif]
